# Letter to Editor: a giant peritoneal loose body in the pelvic cavity

**DOI:** 10.1186/s12957-024-03574-4

**Published:** 2024-11-04

**Authors:** Ke Wu, Qiu-Ling Wang, Wei Ren, Wu-Bin Guo

**Affiliations:** 1 Department of General Surgery, The Affiliated Traditional Chinese Medicine Hospital, Southwest Medical University, Luzhou, China; 2 National Traditional Chinese Medicine Clinical Research Base and Drug Research Center of Integrated Traditional Chinese and Western Medicine, The Affiliated Traditional Chinese Medicine Hospital, Southwest Medical University, Luzhou, China; 3 The Key Laboratory of Integrated Traditional Chinese and Western Medicine for Prevention and Treatment of Digestive System Diseases of Luzhou City, The Affiliated Traditional Chinese Medicine Hospital, Southwest Medical University, Luzhou, China

**Keywords:** Peritoneal loose body, Surgery, Letter to the editor, Abdominal mass, Case

## Abstract

A response to the case report by Zhang et al. and supplement another case of giant peritoneal loose body discovered due to abdominal pain. A 68-year-old man was admitted to the hospital with abdominal pain. CT revealed an ovoid mass in the pelvis measuring approximately 11.5 × 8.6 × 7.4 cm. During laparotomy, yellowish-white mass was identified within the pelvis. Histological examination revealed that the mass was hyalinized fibrous connective tissue with focal calcification. We report an extremely rare and interesting case.

Dear Editor,

We read with interest the article by Zhang et al. [1] which reported a case of peritoneal loose body (PLB) and reviewed relevant literature, summarizing key information about these cases, including gender, age, symptoms, size and weight of the PLBs, and the surgical approaches employed. We would like to supplement this report with a case of our own. The patient is a 68-year-old male who presented with a four-day history of abdominal pain, accompanied by dysuria and painful urination. Upon investigation, an abdominal CT scan revealed the presence of a round, mixed-density mass in the pelvis, with an approximate diameter of 11.5 × 8.6 × 7.4 cm (Fig. 1). The mass exhibited a triangular central region, a high-density border circumferentially, and soft tissue density at the outermost circumference, with clear boundaries. The patient had a history of appendicitis surgery, a two-month course of hepatitis B infection, impaired liver function, and no abnormalities in all other laboratory tests. We hypothesized that the patient’s symptoms were due to the mass. Therefore, a laparotomy was performed. During the surgical procedure, a hard, smooth, yellowish-white mass was identified within the pelvis, exhibiting no discernible adhesion to the abdominal organs. It was found that the visible liver capsule was severely adhered to the omentum, the liver was hard and could not be palpated, the small intestine was mildly adherent with abdominal wall, the appendix at the ileocecal region had been removed, and the remaining pelvic and abdominal organs appeared normal. We removed the mass and separated the adhesions in the small intestine. The cut surface of the mass was gray, hard, and tough, with the presence of hard bone-like tissue observable in the center (Fig. 2). After measuring the mass to be approximately 10 × 8.5 × 7 cm, a histopathological examination was conducted. The pathological diagnosis was hyalinized fibrous connective tissue with focal calcification (Fig. 3), and it was ultimately determined to be a PLB. The patient recovered well and was discharged seven days after the surgery.

We agree that this is a rare case, and apart from the symptoms of abdominal discomfort and compression, PLBs typically do not cause other specific symptoms [2]. The size of PLBs in most literature reports are about 2–10 cm [3], while this PLB is one of the largest so far, with the longest part reaching 10 cm. The patient had a history of appendectomy and intraoperative findings of severe intra-abdominal adhesions. Therefore, we concur with the prevailing view among scholars that PLBs originate from intestinal fat prolapse, fragments of fat tissue from the greater omentum and pancreas, and the free body gradually absorbs proteins in the peritoneum, eventually undergoing saponification, calcification, and fibrogenesis [4].

Preoperative diagnosis of PLBs remains challenging for both clinicians and imaging specialists, as they must be distinguished from intestinal stromal tumors, teratomas, abdominal lymph node calcifications, and tuberculosis granulomas [5]. The mobility of PLBs, which varies with body position, is a key distinguishing feature that can help prevent misdiagnosis [6]. Histopathological examination after surgery remains the gold standard for diagnosis, which can determine the benign or malignant nature of the disease and differentiate it from other diseases, thus providing a basis for subsequent treatment. Histopathology of PLBs usually shows calcified necrosis of adipose tissue with hypocellular fibrolamellar tissue with numerous microcalcifications [7].This case meets the above characteristics.

The accurate diagnosis and subsequent treatment of this disease necessitate the collaboration of multiple disciplines. First, a radiologist is essential for make the most precise diagnosis possible. Following this, a clinician evaluates the patient’s health status and selects an appropriate treatment method. Finally, a pathological examination is conducted to ascertain the nature of the pathology. Given the rarity of these cases in clinical practice, it is anticipated that future researchers will compile comprehensive patient medical histories and ancillary examinations, conduct in-depth molecular analyses of PLBs, elucidate the underlying pathogenesis, and provide evidence to enhance the diagnosis and treatment of PLBs.

**Fig. 1:**
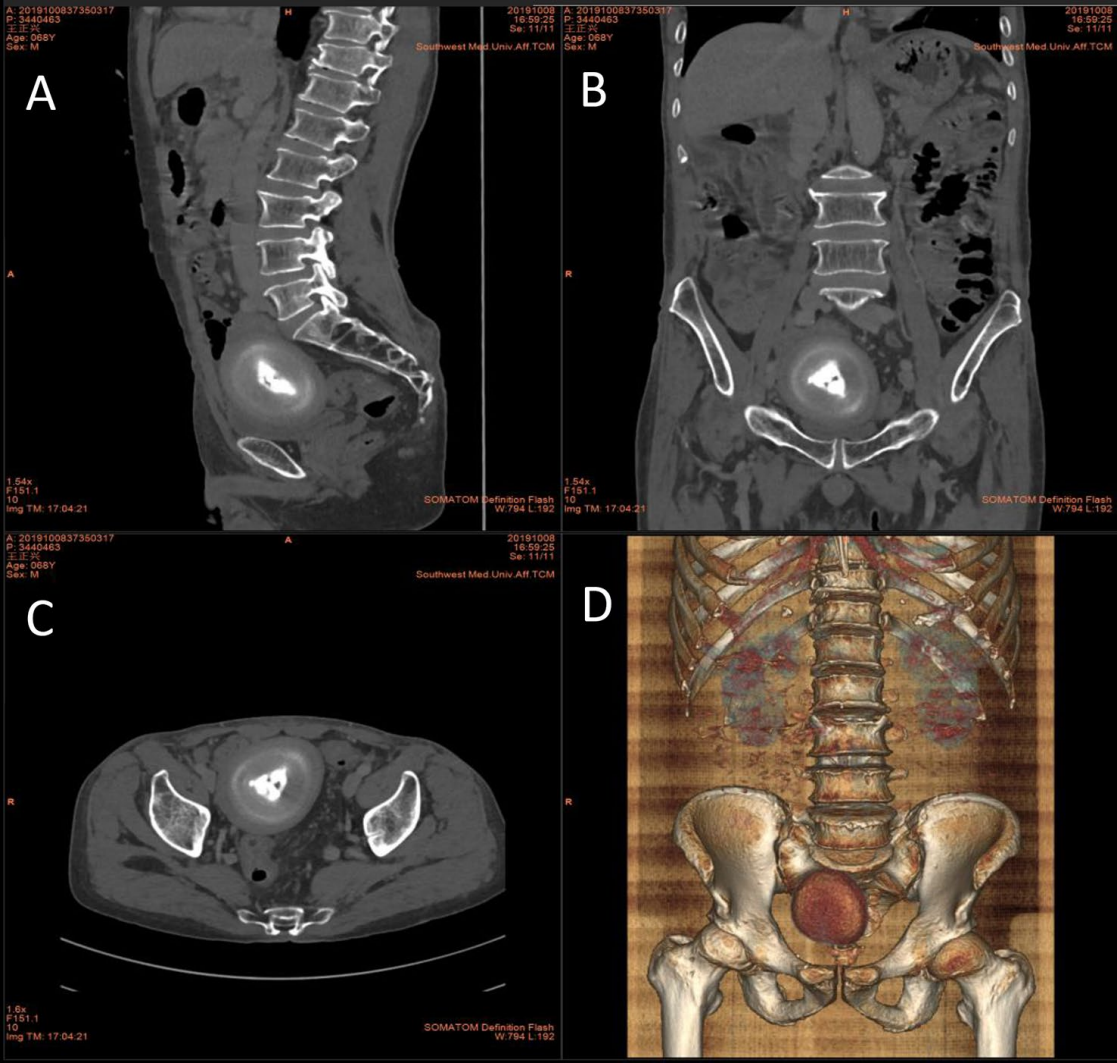
CT scan: A rounded, mixed-density mass was founded in the pelvis, measuring approximately 11.5 × 8.6 × 7.4 cm. **A**: sagittal; **B**: coronal; **C**: transverse; **D**: 3D image reconstruction

**Fig. 2:**
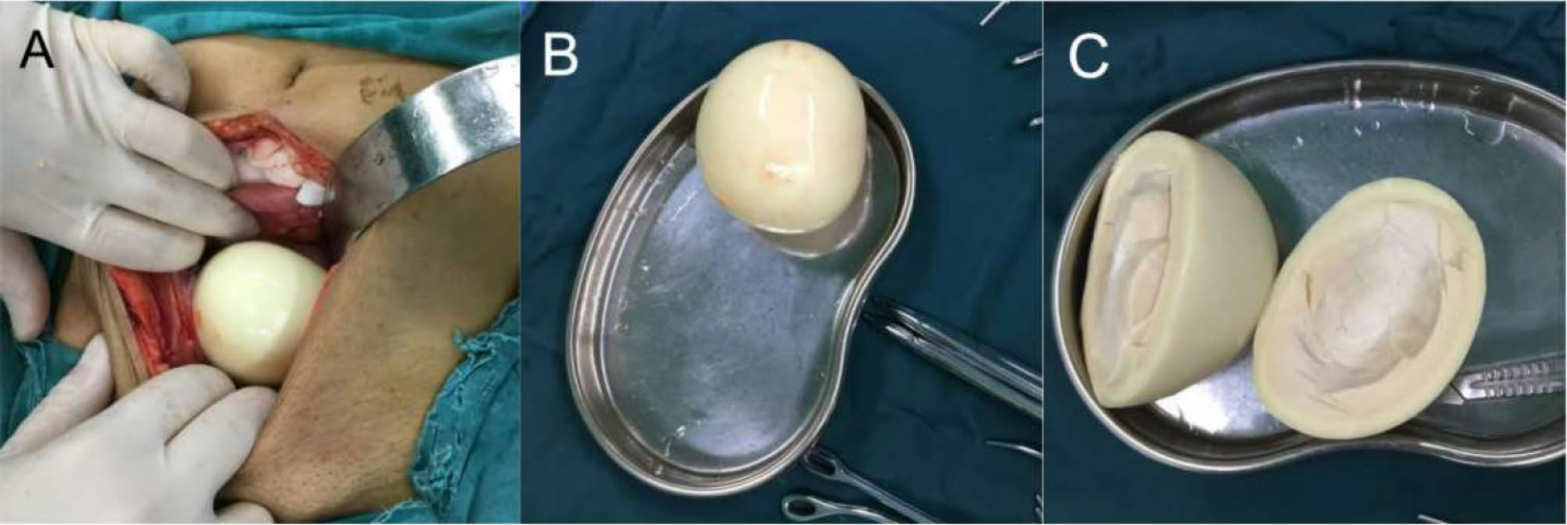
**A**: Mass during surgery; **B**: A hard, smooth, yellowish-white mass; **C**: The cut surface was grayish, solid, and tough, with visible hard bone-like tissue at the center

**Fig. 3:**
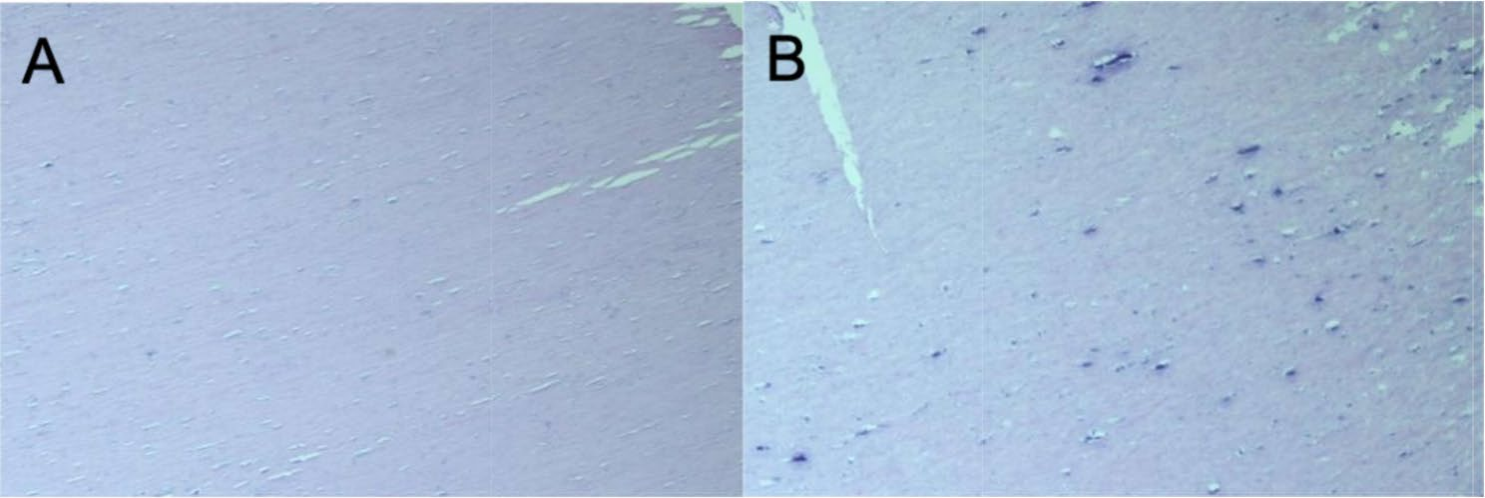
**A** Fibrous connective tissue with hyaline degeneration; **B**: Focal calcification in the center of the mass. (HE×40)

## Data Availability

No datasets were generated or analysed during the current study.

## Abbreviations

PLB: Peritoneal loose body
