# Correction: Letter to editor: a giant peritoneal loose body in the pelvic cavity

**DOI:** 10.1186/s12957-025-03688-3

**Published:** 2025-02-01

**Authors:** Ke Wu, Qiu-Ling Wang, Wei Ren, Wu-Bin Guo

**Affiliations:** 1 Department of General Surgery, The Affiliated Traditional Chinese Medicine Hospital, Southwest Medical University, Luzhou, China; 2 National Traditional Chinese Medicine Clinical Research Base and Drug Research Center of Integrated Traditional Chinese and Western Medicine, The Affiliated Traditional Chinese Medicine Hospital, Southwest Medical University, Luzhou, China; 3 Key Laboratory of Integrated Traditional Chinese and Western Medicine for Prevention and Treatment of Digestive System Diseases of Luzhou City, The Affiliated Traditional Chinese Medicine Hospital, Southwest Medical University, Luzhou, China

**Correction: World J Surg Onc 22**,** 289 (2024).**

10.1186/s12957-024-03574-4

Following publication of the original article [1], the author reported that the affiliations should be updated

From

Ke Wu**1**,**2**, Qiu-Ling Wang1†, Wei Ren2 and Wu-Bin Guo**3**

To

Ke Wu**1**, Qiu-Ling Wang1†, Wei Ren2 and Wu-Bin Guo**1**,**3**

This has been updated above and original article has been updated.
